# 可手术的非小细胞肺癌患者贫血的发生和预后的生存分析

**DOI:** 10.3779/j.issn.1009-3419.2010.07.12

**Published:** 2010-07-20

**Authors:** 秋华 邓, 海虹 杨, 鑫 张, 汉章 陈, 源 邱, 丹萍 温, 信国 熊, 炜 王, 建行 何

**Affiliations:** 510120 广州，广州医学院第一附属医院广州呼吸疾病研究所 Institute of Respiratory Diseases, the First Affiliated Hospital of Guangzhou Medical College, Guangzhou 510120, China

**Keywords:** 肺肿瘤, 贫血, 生存率, 预后, Lung neoplasms, Anemia, Survival rate, Prognosis

## Abstract

**背景与目的:**

非小细胞肺癌（non-small cell lung cancer, NSCLC）患者可发生贫血, 然而贫血是否为NSCLC患者预后的独立因素之一, 仍存在争议, 故本文研究可手术的NSCLC患者贫血的发生情况和影响预后的因素。

**方法:**

对广州医学院第一附属医院2000年1月-2008年12月住院的1 018例NSCLC患者进行回顾性分析, 分析NSCLC手术患者贫血的发生情况以及影响预后的因素。

**结果:**

患者术前贫血发生率为252/1 018（24.1%）, 无贫血患者总生存时间为（2 425.98±50.03）天; 贫血患者的总生存时间是（2 107.15±93.86）天, 有无贫血患者的总生存时间存在明显差异（*P*=0.001）。*Kaplan-Meier*生存分析显示Ⅰ期NSCLC有无贫血患者的生存时间存在显著差异（*P* < 0.001）, 但是在Ⅱ期（*P*=0.310）和Ⅲa期（*P*=0.458）患者中, 生存时间无明显差异, 且Ⅰ期、Ⅱ期和Ⅲa期的NSCLC患者累积生存时间存在统计学差异。*Cox*回归分析结果显示NSCLC患者的TNM分期、性别、肿瘤大小、是否淋巴结转移均与预后显著相关。

**结论:**

贫血可以作为NSCLC可手术患者的预后相关因素之一, 但在Ⅰ期NSCLC患者中是独立的预后因素。

## Abstract (en)

**Background and objective:**

The patients with non-small cell lung cancer (NSCLC) might contract anemia, however, whether anemia is one of the independent prognostic factors to the patients with NSCLC is still controversial. So the aim of this study is to investigate the correlation between anemia and overall survival (OS) in patients with NSCLC.

**Methods:**

1 018 patients with operable NSCLC were retrospectively analyzed in our hospital from January 2000 to December 2008.

**Results:**

The occurrence of anemia before operation was 252/1 018 (24.1%). The OS in NSCLC patients without anemia was (2 425.98±50.03) days, and the OS in patients with anemia was (2 107.15±93.86) days. There was significant difference in the OS between them (*P*=0.001). The patients with anemia in stage Ⅰ had shorter survival time than those without anemia (*P* < 0.001). But there was no difference in other stage patients. TNM stage, gender, tumor size and lymph nodes metastasis were correlated with OS using *Cox* regression analysis.

**Conclusion:**

Anemia is correlated with survival in operable NSCLC patients. Moreover, it is an independent prognostic factor in NSCLC patients with stage Ⅰ.

贫血是恶性肿瘤治疗中仅次于消化道反应的较为常见的并发症。首先，恶性肿瘤本身可以导致贫血的发生；其次，出血等并发症以及放化疗等治疗方法也可导致贫血。贫血可以损害患者各个器官系统功能，也可降低患者的生存质量，最终影响肿瘤的治疗疗效而危及患者的生命^[1, 2]^。目前肺癌是恶性肿瘤死亡的首位原因，其中80%是非小细胞肺癌（non-small cell lung cancer, NSCLC），但是NSCLC患者肿瘤相关性贫血和预后之间关系的研究仍存在争议。因此，本研究通过对广州医学院第一附属医院1 018例具有完整生存资料的NSCLC患者的回顾性分析，以探讨肺癌相关性贫血的发生情况，以及和生存预后的相关性。

## 1 资料与方法

### 1.1 一般资料

选取广州医学院第一附属医院2000年1月-2008年12月NSCLC术前患者1 018例。所有病例均有病理证实，无溶血及明显的营养不良等，无肝肾功能不全。

### 1.2 检测方法与数据收集

清晨抽取静脉血2 mL于抗凝管中，日本Sysmex公司的血细胞检测分类仪检测血红蛋白（Hb）。通过电话随访生存。

### 1.3 贫血分级标准

贫血诊断按照《内科学》的贫血诊断标准，即贫血是指Hb < 120 g/L（男）或Hb < 110 g/L（女）。

### 1.4 临床分期标准

根据国际抗癌联盟（International Union Against Cancer, UICC）2008版肺癌分期手册进行TNM临床分期。

### 1.5 统计学分析

采用SPSS 17.0进行统计学分析。分析贫血和NSCLC患者临床特征之间的相关性采用χ^2^检验，应用*Kaplan-Meier*法分析贫血和患者生存预后的关系，建立*Cox*模型进行患者预后相关的多因素回归分析，以*P* < 0.05为差异有统计学意义。

## 2 结果

### 2.1 一般临床情况

选取2000年1月-2008年12月NSCLC患者1 018例，其中男724例，女294例；年龄30岁-82岁。Ⅰ期患者500例，Ⅱ期患者249例，Ⅲa期患者269例（表 1）。单纯手术患者571例，术后辅助化疗病人446例。按治疗前Hb值的不同将患者分为两组，即男Hb≥120 g/L或女≥ 110 g/L为无贫血组，男Hb < 120 g/L或女 < 110 g/L为贫血组。在不同年龄（*P*=0.014）、肿瘤大小（*P*=0.042）、病理类型（*P*=0.045）、血清白蛋白（*P*=0.002）和化疗（*P*=0.001）组患者中，贫血的发生率具有显著的统计学差异。

**1:** 1 018例非小细胞肺癌患者临床特征 Demographic and clinical characteristics of 1 018 NSCLC patients

	Patients (*n*)	Withoutanemia (*n*, %)	With anemia (*n*, %)	*P*
Gender				
Male	724	539 (70.0) 185 (74.6)		
Female	294	231 (30.0) 63 (25.4)		0.165
Age/yr				
< 70	822	635 (82.5) 187 (75.4)		
≥70	196	135 (17.5) 61 (24.6)		0.014
Tumor size				
T≤3 cm	272	218 (28.3) 54 (21.8)		
T > 3 cm	745	551 (71.7) 194 (78.2)		0.042
Lymph nodes metastasis				
Yes	265	199 (25.8)	66 (26.6)	
No	753	571 (74.2)	182 (73.4)	0.810
Clinical stage				
Ⅰ	500	381 (49.5)	119 (48.0)	
Ⅱ	249	188 (24.4)	61 (24.6)	
Ⅲa	269	201 (26.1)	68 (27.4)	0.900
Histology				
Squamous cell carcinoma	294	208 (27.0)	86 (34.7)	
Adenocarcinoma	522	448 (56.3)	119 (48.0)	
Others	171	128 (16.6)	43 (17.3)	0.045
Albumin				
< 35	212	143 (18.6)	69 (27.8)	
≥35	806	627 (81.4)	179 (72.2)	0.002
Chemotherapy				
Yes	446	361 (46.9)	85 (34.3)	
No	571	408 (53.1)	163 (65.7)	0.001

### 2.2 生存分析

将贫血作为预后因素，分析其对NSCLC患者生存的影响。首先将患者按有无贫血分组：无贫血患者的生存期为（2 425.98±50.03）天，贫血患者的生存期为（2 107.15±93.86）天，提示无贫血和贫血患者的生存期存在显著差异（*P*=0.001）（图 1）。

**1:**
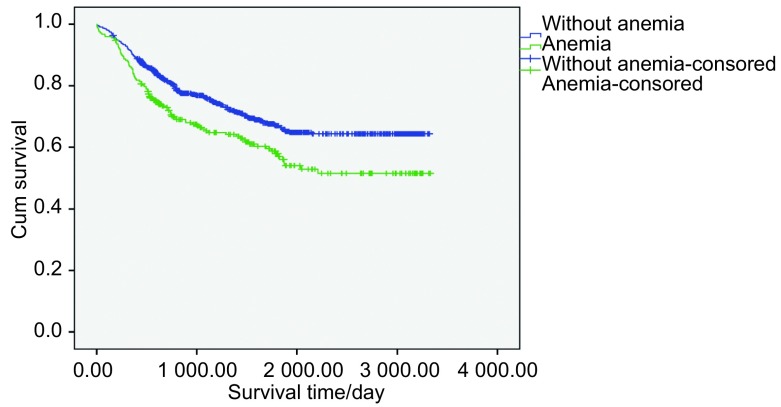
无贫血组和贫血组患者*Kaplan-Meier*累计生存时间曲线 *Kaplan-Meier* cumulative survival time curves of without anemia group and anemia group patients

将患者按肿瘤TNM分期分为3个亚组，在不同肿瘤分期亚组中进行贫血的发生和生存的相关性分析。结果提示，NSCLC患者肿瘤分期和贫血的发生存在明显相关性（*P*=0.002），且在Ⅰ期肺癌患者中，无贫血患者生存期（2 686.10±62.27）天，贫血患者生存期为（2 186.37± 123.12）天，Ⅰ期NSCLC患者无贫血者的生存预后显著好于贫血患者（*P* < 0.001）。但是在Ⅱ期（*P*=0.310）和Ⅲa期（*P*=0.458）NSCLC患者中，有无贫血患者的生存时间并无统计学差异（图 2）。Ⅰ期、Ⅱ期和Ⅲa期的NSCLC患者累计生存率存在显著差异（*P* < 0.001）（图 3）。

**2:**
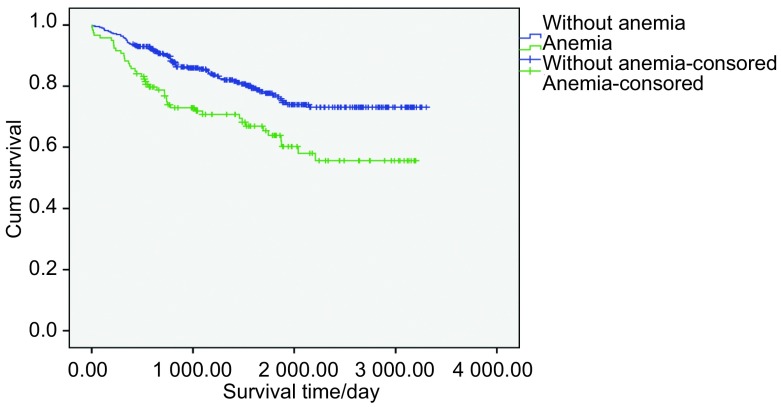
Ⅰ期无贫血组和贫血组患者*Kaplan-Meier*累计生存时间曲线 *Kaplan-Meier* cumulative survival time curves of without anemia group and anemia group in stage Ⅰ patients

**3:**
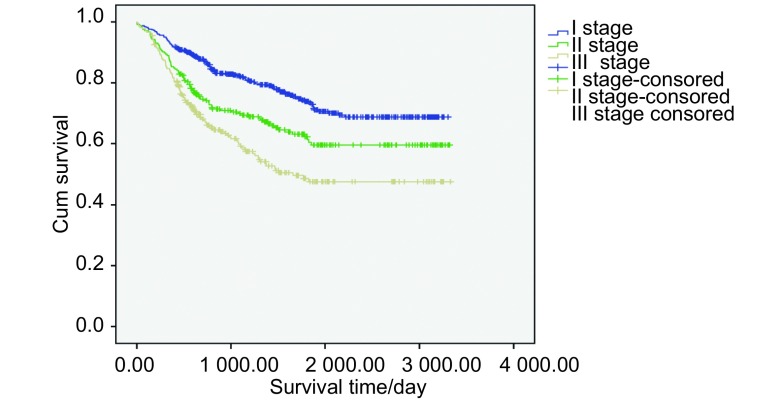
Ⅰ期、Ⅱ期和Ⅲa期患者*Kaplan-Meier*累计生存时间曲线 *Kaplan-Meier* cumulative survival time curves of stage Ⅰ, stage Ⅱ and stage Ⅲ group patients

对1 018例NSCLC患者进行单因素*Kaplan-Meier*分析，结果显示不同性别（*P*=0.002）、肿瘤大小（*P* < 0.001）、和临床分期（*P* < 0.001）、有无淋巴结转移（*P* < 0.001）、低白蛋白（*P*=0.017）、贫血（*P*=0.001）的患者有明显的生存差异，但是不同年龄（*P*=0.125）、病理类型（*P*=0.650）和化疗（*P*=0.300）的患者无明显生存差异。

### 2.3 影响肺癌患者预后的多因素回归分析

将患者的性别、贫血状况、肿瘤大小、有无淋巴结转移、临床分期和白蛋白情况等单因素引入*Cox*回归模型。结果显示只有TNM分期、性别、肿瘤大小、是否淋巴结转移和预后显著相关，而白蛋白情况、有无贫血和有无化疗与患者预后无显著相关性（表 2）。

**2:** 影响1 018例NSCLC患者生存期的预后因素(*Cox*回归分析） Prognostic factors for surival in patients with 1 018 NSCLC patients (*Cox* regression model)

Characteristics	B	*P*	Hazard ratio (HR)	95% confidence interval	
				Lower	Upper
Gender (Female; Male)	0.488	0.001	1.629	1.221	2.174
Tumor size (≥3 cm; < 3 cm)	-0.312	0.042	0.732	0.542	0.988
Lymph node metastasis (Yes; No)	-0.508	0.001	0.602	0.464	0.781
Clinical stage (Stage Ⅲa-Ⅰ)	-0.375	0.009	0.688	0.519	0.910
Albumin (Low; Normal)	-0.171	0.229	0.843	0.637	1.114
Anemia (No; Yes)	0.171	0.565	1.187	0.662	2.130

## 3 讨论

贫血是恶性肿瘤患者的常见合并症，大约50%的肿瘤患者发生贫血，而在晚期肿瘤及接受化放疗的患者中，贫血的发生率则高达90%^[3]^。肿瘤相关性贫血越来越受到广泛的关注，特别是贫血与生活质量及治疗结果有关。本研究中已排除了出血、溶血、明显的营养不良和肝肾功能不全的情况，因此患者的贫血可以认为是癌性贫血或癌症治疗相关性贫血。本研究中贫血的发生率为24.1%。Ludwig等^[4]^报道，1 898例欧洲肺癌患者癌症相关性贫血的发生率约为77%，治疗相关性贫血为50%左右。本研究中的患者均为初诊病人，未经过放化疗治疗，因此无放化疗相关性贫血，癌症相关性贫血（非治疗相关性）的发生率和该报道类似，且我们发现在不同年龄、肿瘤大小、病理类型、血清白蛋白和化疗组患者中，贫血的发生率具有显著的统计学差异，与顾琳萍的研究^[5]^中放化疗组贫血构成比具有明显差异基本一致。这因为放化疗本身就可以引起患者贫血，而病理状况和血清白蛋白水平影响NSCLC患者贫血的治疗效果，进一步加重NSCLC患者的贫血，而≥70岁的NSCLC患者比 < 70岁的患者更易发生贫血，估计是与老年人对治疗的耐受性差有关。

贫血直接影响患者的生活质量，甚至可降低患者的认知能力，并且可引起肿瘤细胞内乏氧，造成肿瘤药物治疗的耐药而影响疗效。因此，血红蛋白水平可能是影响肺癌患者生存期的预后因素之一，多项研究报道^[6, 7]^发现贫血可作为与肿瘤患者的生存期相关的一个独立因素，纠正贫血可以改善肿瘤患者的转归^[8-13]^。但是目前仍有不一致的报道^[14]^。我们通过研究1 018例NSCLC患者的生存数据，分析了贫血和NSCLC患者生存预后的相关性。通过分层分析NSCLC患者的生存状态，发现本研究中的肺癌患者中，无贫血患者的生存时间明显长于贫血患者的生存时间（*P*=0.001），与文献的结论^[15]^相似。对患者按TNM分期进行分层分析，发现Ⅰ期NSCLC无贫血患者生存明显好于贫血患者，但是在其它分期患者中无明显差异。我们的结果显示Ⅰ期NSCLC患者血红蛋白水平与肺癌生存期显著相关，是独立的预后因素之一，而在Ⅱ期和Ⅲa期NSCLC患者血红蛋白水平与肺癌生存期无相关性，估计因为影响Ⅱ期和Ⅲa期NSCLC患者预后的因素比较多，例如有淋巴结的转移、化疗等影响，Ⅰa期可以排除这些方面的影响。

本研究在单因素分析中显示临床分期与生存时间有关，这与文献报道一致^[16]^。并且无淋巴结转移、正常白蛋白水平、无贫血的生存率较有淋巴结转移、低白蛋白、贫血的患者明显增高，提示临床医生积极纠正NSCLC患者的血红蛋白和白蛋白水平会有利于改善NSCLC患者的预后。我们进一步建立了*Cox*模型，将上述6个因素作为独立变量纳入此多元回归方程。结果发现在上述6个因素中，非小细胞肺癌患者的TNM分期、性别、肿瘤大小、是否淋巴结转移是影响其预后的独立因素。性别是独立预后因素之一，考虑其原因可能与我们选择的病例中大部分是男性患者，存在性别分布不均有关。但是进行多因素分析时，并未发现贫血是独立的预后因素，因此贫血可能只是影响NSCLC患者预后的相关因素。

总之，贫血是NSCLC可手术患者的预后相关因素之一，但在Ⅰ期NSCLC患者中是独立的预后因素。
